# Prevalence and psychometric screening for the detection of major depressive disorder and post-traumatic stress disorder in adults injured in a motor vehicle crash who are engaged in compensation

**DOI:** 10.1186/s40359-018-0216-5

**Published:** 2018-02-21

**Authors:** Rebecca Guest, Yvonne Tran, Bamini Gopinath, Ian D. Cameron, Ashley Craig

**Affiliations:** 1 John Walsh Centre for Rehabilitation Research, Sydney Medical School-Northern, The University of Sydney, Kolling Institute of Medical Research, St Leonards, NSW Australia; 2 Key University Centre for Health Technologies, University of Technology, Broadway, Sydney, NSW Australia; 3 Sydney Medical School-Northern, Kolling Institute of Medical Research, The University of Sydney, Royal North Shore Hospital, Corner Reserve Road & Westbourne Street, St Leonards, NSW 2065 Australia

**Keywords:** Motor vehicle accident, Depression, Post-traumatic stress disorder, DASS-21, IES-r, Compensation, Physical injury, MVA, MVC, PTSD

## Abstract

**Background:**

Physical injury and psychological disorder following a motor vehicle crash (MVC) is a public health concern. The objective of this research was to determine rates of major depressive disorder (MDD) and post-traumatic stress disorder (PTSD) in adults with MVC-related injury engaged in compensation, and to determine the capacity (e.g. sensitivity and specificity) of two psychometric scales for estimating the presence of MDD and PTSD.

**Methods:**

Participants included 109 adults with MVC-related injury engaged in compensation during 2015 to 2017, in Sydney, Australia. The mean time from MVC to baseline assessment was 11 weeks. Comprehensive assessment was conducted at baseline, and the Depression Anxiety Stress Scales (DASS-21) and the Impact of Event Scale-Revised (IES-R) were administered to determine probable MDD and PTSD. An online psychiatric interview, based on Diagnostic and Statistical Manual for Mental Disorders (DSM-5), was used to diagnose actual MDD and PTSD, acknowledged as gold standard diagnostic criteria. One-way multivariate analyses of variance established criterion validity of the DASS-21 and IES-R, and sensitivity and specificity analyses were conducted to determine the most sensitive cut-off points for detecting probable MDD and PTSD.

**Results:**

Substantial rates of MDD (53.2%) and PTSD (19.3%) were found. The DASS-21 and IES-R were shown to have excellent criterion validity for detecting MDD and PTSD in injured participants. A range of cut-off points were investigated and shown to have acceptable sensitivity and specificity for detecting MDD and PTSD in an injured population engaged in compensation. The preferred cut-off points based on this study are: to detect MDD, a DASS-21 total score of 30 and/or a DASS-21 depression score of 10; to detect PTSD, IES-R scores of 33–40 and/or a DASS-21 anxiety score of 7–8.

**Conclusions:**

Major psychological disorder is prevalent following a MVC. Results suggest the DASS-21 and IES-R are suitable for use in clinical/compensation settings to detect probable MDD and PTSD soon after a MVC in physically injured people engaged in compensation. These results provide positive direction in the public health arena for improving mental health outcomes.

**Trial Registration:**

Clinical Trials registration number: ANZCTR - ACTRN12615000326594 (9th April 2015).

## Background

Physical injury associated with motor vehicle crashes (MVC) is a principal cause of morbidity and mortality [1, 2] and viewed as a major public health crisis. Disability arising from MVCs is estimated to escalate globally unless road safety and management of injury-related impairment are improved [3, 4]. Rates of disability associated with MVCs are high, with almost 60% of car occupants who sustain physical injury experiencing significant incapacity and health problems [5, 6], and associated economic and compensation costs are substantial [7]. For example, in Australia the cost associated with MVCs was approximately $17b or 2.3% of gross domestic product in recent years [7].

Psychological disorder is an additional risk and burden following a MVC [3, 4, 8–10]. A recent meta-analysis revealed psychological distress to be substantially elevated following a MVC in people with physical injuries such as whiplash, traumatic brain injury (TBI) and spinal cord injury (SCI), resulting in greater risk of psychological disorder [3]. Major depressive disorder (MDD) and post-traumatic stress disorder (PTSD) are common disorders associated with a MVC [4, 11–13]. Rates of MDD and PTSD have been shown to be high up to 12 months post-MVC, with for example, almost 30% of people at risk of MDD after sustaining TBI or SCI [12, 14]. Recent prospective research found 1 in 2 persons suffered elevated rates of depression and PTSD soon after a MVC and elevated rates were still present 12 months later [15]. In a systematic review, median occurrence of PTSD in people sustaining physical injury in a MVC was found to be around 30% 1 month post-MVC, with a declining trend at 12 months to 15% [13]. In prospective research, drivers and passengers who had sustained injury in a MVC had significantly elevated levels of traumatic distress of around 30% (i.e. probable PTSD) within 4 weeks of the MVC, declining to a probable PTSD rate of 20% 6 months after the MVC [16].

Research indicates that lodging a claim and seeking compensation following a MVC increases risk of psychological distress in claimants [17–19]. For example, in a large sample of adults engaged in compensation following injury in a MVC, mood and anxiety were predicted by factors such as catastrophizing styles of thinking about their chronic pain and life, and dissatisfaction about their claim process [18]. Additionally, the presence of psychological disorder during compensation was found to be significantly associated with higher MVC-related costs, and at least double the time to claim completion, factors that will likely increase risk of psychological disorder after the compensation process [20].

A range of psychometric screens and measures have been used to assess MDD and PTSD following a MVC [3, 13, 15, 16]. Structured diagnostic interviews, such as the Structured Clinical Interview for DSM are based on criteria from the DSM (SCID; http://www.scid4.org/) or International Classification of Diseases (ICD; http://www.who.int/classifications/icd), have been used in previous research as gold standard strategies for diagnosing psychological disorders [12, 21]. Arguably however, diagnostic interviews are less desirable for use in public health/compensation settings because they increase assessment time substantially and involve complex decision pathways by specifically trained professionals. These factors combined also make them an expensive assessment strategy to use in research with large populations. The compensation setting involves large populations of physically injured MVC claimants, managed by time restricted case managers not trained in clinical diagnoses or assessments. This necessitates the use of easily administered and time efficient psychometric tools to determine outcomes such as psychological distress that could be easily understood by these case managers. Consequently, psychometric self-report instruments are often used for estimating probable rates of psychological disorder, even though there remains uncertainty about the capacity of these tools to detect disorders like MDD and PTSD. Problems of detection in psychometric screens include the propensity to produce false positives (i.e. those incorrectly diagnosed with MDD or PTSD) and false negatives (those who have MDD and/or PTSD but it is not detected) [22]. This introduces the concept in public health of sensitivity and specificity [22, 23]. Sensitivity is the probability that a test result will be positive when the disorder/disease is present (true positive rate), whereas specificity is the probability that a test result will be negative when the disorder/disease is not present (true negative rate). If a diagnostic strategy has limited sensitivity and specificity, then public health and clinical consequences are problematic. For example, health costs will be greatly inflated and resources stretched if interventions are delivered to those incorrectly diagnosed. Likewise, failing to detect a disorder will result in human suffering and also result in higher costs if the person subsequently deteriorates.

The authors believe two scales that have been extensively used for detecting psychological disorder have promise for use in compensation settings and were therefore selected to investigate their capacity to detect MDD and PTSD. The first scale, the Depression Anxiety Stress Scales (DASS) [24, 25] was chosen as the preferred screen for MDD rather than a more specific screen like the self-report Patient Health Questionnaire-9 (PHQ-9) [26] because the DASS-21 is widely used in clinical settings and it has substantial data available on its validity and reliability. It provides broader information about mood, anxiety and levels of stress from 21 items that presents twice the amount of information than the PHQ-9 on aspects not only on symptoms of mood, but also questions physical symptoms of anxiety, for example, “I experience trembling”, “I find myself getting agitated” and “I experience breathing difficulty (e.g. excessively rapid breathing)”. Further, the DASS-21 has been used for assessing mood, anxiety and stress in populations such as injury, back pain, SCI and depressed people in the community [16, 23, 27, 28]. For example, DASS-21 was shown to be suitable for use in an occupational health care setting in which it was used to detect possible psychological disorder in employees with mental health problems [23]. A cut-off score of 12 (sensitivity 91%, specificity 46%) on the depression domain was concluded best to detect MDD [23]. DASS-21 depression domain (sensitivity 86% specificity 64%) was shown to be a sensitive instrument for detecting depression in SCI [28]. Research that used the DASS-21 to estimate depression in the community concluded an optimal cut-off was a total score of 36 (sensitivity 80.8%, specificity 75.4%) [29]. The DASS-21 has not been used to detect MDD in adults who have experienced MVC-related physical injury and engaged in compensation. A recent meta-analysis on psychological distress following MVC injury has provided information on a large range of measures used to measure distress [3]. However most of these measures in our view are not as appropriate or useful as the DASS-21 as they either take too long to administer or they are specifically mood questionnaires, or they just focus on anxiety [3, 12, 15].

The second scale, the Impact of Event Scale-Revised (IES), [30, 31] has been widely used with people experiencing trauma (e.g. returned veterans and victims of a MVC) and has been shown to be a valid measure of trauma distress in MVC survivors [32, 33]. Based on norms, a total score of 33 is believed to represent probable PTSD [33]. The IES-R was used to detect PTSD in adults experiencing injury after a MVC, recruited from emergency departments in Europe [15]. However, a cut-off score based on only two of the three IES-R domains (intrusion and avoidance) was used, resulting in less items in the scale, and a low cut-off score ≥ 26 as indicating probable PTSD [15]. This cut-off score is therefore not appropriate if one uses the total IES-R scale (i.e. intrusion, avoidance and hyperarousal) because not all items in the scale have been included in the cut-off calculation. None of the above papers have reported sensitivities or specificities related to the cut-off scores employed. The findings from this study will address this limitation.

The aims of the current study were: (i) given the lack of published information, the prevalence of MDD and PTSD was calculated in a sample of adults who have experienced a MVC and engaged in compensation; (ii) to investigate the criterion validity of the DASS-21 and IES-R for measuring MDD and PTSD in adults physically injured in a MVC and engaged in compensation; (iii) determine the capacity (e.g. true positive and true negative rates) of the two psychometric scales for detecting MDD and PTSD. This will involve the exploration of the sensitivity and specificity of various cut-off points for these two scales, and whether optimal cut-off points can be determined by comparing results with a gold standard criterion, that is, diagnosis based on DSM-5 criteria for MDD and PTSD.

## Method

### Recruitment and participants

In New South Wales (NSW) Australia, compensation following a MVC is available under a compulsory third party (CTP) insurance scheme. This insurance is compulsory for the owners of all motor vehicles. People are eligible to lodge a claim if they are injured as a result of the MVC and, in NSW, are not at fault (with some limited exceptions for at fault drivers where they can claim up to $5000 Australian for injury related costs) [20]. Victoria has a no fault CTP scheme, where compensation can be given regardless of fault status. If eligible, the injured person can make a claim for a range of benefits including medical treatment and rehabilitation costs, care costs, economic losses, as well as payments for pain and suffering. Claimants must have reported the accident and injuries within 48 h of the road crash, and lodge the CTP claim within 6 months from the date of the crash.

This study is part of a larger study investigating brief psychological interventions aimed at reducing the psychological distress of those physically injured in a MVC and engaged in compensation. Recruitment involved an opt-in process in which claimants meeting inclusion/exclusion criteria were contacted by an insurance company case manager for their interest in participating in the research, followed by the researcher telephoning the potential participant to discuss the research further. Information sheets and consent forms were then emailed to those people who indicated willingness to participate. Inclusion criteria consisted of (i) MVC survivors aged 18 years or over who have lodged a compensation claim within 3–4 months of the MVC (i.e. we wanted to reduce chances of recruiting claimants who had developed a chronic psychological disorder, arguably more likely by 5–6 months post road crash), and (ii) English speaking. Exclusion criteria consisted of sustaining catastrophic or complex injuries, which according to NSW guidelines defined by the icare lifetime care authority, include injuries such as spinal cord injury, amputation, blindness, multiple fractures and internal damage requiring extended hospitalization, or severe traumatic brain injury [34].

Altogether, 411 persons who met inclusion/exclusion criteria were approached by case managers, with 252 (61.3%) indicating willingness to discuss the study with the researchers. After discussion and reading the information sheet, 109 elected to participate in the study providing written consent, representing a recruitment rate of 43.2% (109/252). Reasons for non-consent included i) assistance not required, ii) not enough time to devote to the intervention, iii) too much pain, iv) advice from lawyer not to receive assistance. The 109 adults who consented to participate were recruited through three compulsory third party (CTP) insurers (two in New South Wales, Australia and one in Victoria, Australia), over a period of almost 2 years (from July 2015 to May 2017). Case managers in each of the insurer companies introduced the research to those meeting inclusion criteria, and the names, telephone number and email address of those who were interested were sent to the researchers to discuss the research in more detail and gain consent. Once consent was achieved, the participant was randomized into the study.

Socio-demographic, injury and psychological characteristics are shown in Table 1. Full compliance with the Code of Ethics of the World Medical Association occurred when conducting this study and research ethics approval was granted by the local institutional human research ethics committee. Written consent was obtained prior to participation in the study.

**Table 1:** Socio-demographic and injury characteristics of the 109 participants

Characteristics	Participants
Age: mean years (SD min max)	45.2 (14.9, 18–82)
Female: n (%)	68 (62.4%)
BMI: median mean (SD)	26.8 29.4 (14.7)
Weeks since MVC: mean weeks (SD)	11.4 (9.0)
Days in hospital: mean (median, SD)	1.05 (0, 2.7)
Education	
10 years n (%)	21 (19.3)
12 years n (%)	14 (12.9)
Technical n (%)	25 (22.9)
University n (%)	49 (44.9)
Marital status	
Married/defacto n (%)	57 (52.3)
Single: n (%)	26 (23.8)
Widowed/separated/divorced: n (%)	26 (23.9)
Role in MVC	
Driver and passenger n (%)	83 (76.2)
Motorbike rider n (%)	13 (11.9)
Bicyclist n (%)	8 (7.3)
Pedestrian n (%)	5 (4.6)
Pre-MVC work status	
Full time/Part-time n (%)	84 (77.1)
Student n (%)	1 (0.9)
Pensioner n (%)	16 (14.7)
Unemployed (%)	8 (7.3)
Injury type/location^a^	
Neck n (%)	34 (31.2)
Shoulder n (%)	7 (6.4)
Arm n (%)	7 (6.4)
Upper back n (%)	13 (11.9)
Lower back n (%)	15 (13.8)
Leg n (%)	18 (16.5)
Head n (%)	5 (4.6)
Chest/abdomen n (%)	6 (5.5)
Perceived danger in MVC (median)	3
None or small: n (%)	47 (43.1)
Moderate, great, overwhelming: n (%)	62 (56.9)
Pain intensity mean (SD)	6.8 (2.4)
Treated by psychologist/psychiatrist pre-MVC: n (%)	32 (29.4)
Psychiatric medications pre-MVC: n (%)	28 (25.7)

^a^4 missing values (3.7%)

### Study design and procedure

This study is part of a multi-site three-arm randomized controlled trial (RCT) with two active interventions and one active waitlist control. The aim of the RCT is to determine the efficacy of cognitive behavior therapy (CBT) to prevent/reduce rates of MDD and PTSD in those physically injured MVC survivors engaged in compensation. Full details of the RCT can be found elsewhere [35]. The trial registration number is ACTRN12615000326594. All participants are being assessed four times, that is, a baseline assessment generally within 4 months of the MVC (people can often lodge claims more than 2 months post-MVC); assessment 2 occurring immediately after the 10 week intervention, that is 10 weeks post-baseline; assessment 3 occurring 6 months post-baseline and assessment 4 occurring 12 months post-baseline assessment. However, the data presented in this paper was only drawn from baseline assessment. All participants were directed to a secure online site to complete the baseline/pre-intervention assessment, including the DASS-21, IES-R and DSM criteria. Those who did not have access to the internet were mailed the complete assessment with a return mail envelope. All participants were also telephoned to ensure they understood assessment instructions [35].

### Assessment

Demographic assessments included age, sex, education, pre-MVC work status, and marital status. BMI was calculated using the formula: [weight/(height)^2^]. MVC details included the role of the participant in the accident, days spent in hospital after the crash, and self-reported principal injury type/location. Perceived danger of death during the road crash was also assessed on a 5-point Likert scale (1 = none, 2 = small, 3 = moderate, 4 = great, 5 = overwhelming). To establish self-reported pre-MVC psychological morbidity, participants were asked whether they had ever been treated by a psychiatrist or psychologist for low mood or anxiety (yes or no), and whether they had ever been prescribed medication for low mood or anxiety (yes or no). Pain intensity at the time of interview was measured using an 11-point Likert scale (0 = no pain and 10 = worst pain ever). Research shows numerical pain rating scales have good test–retest reliability and validity [36].

The DASS-21 is a 21-item scale providing an overall assessment of general psychological distress as well as three domains: depressive mood, anxiety and perceptions of stress [24, 25]. Participants completed 21 4-point Likert items (0–3) assessing self-reported distress over the past week. Higher scores indicate elevated distress. Scores are calculated by summing items [25], and then, in accordance with the original DASS-42 the scores were multiplied by 2 (ranging from 0 to 126) [25]. The DASS-21 has sound psychometric properties including acceptable internal reliability and validity [24]. Based on DASS-21 norms, a total score of 32 is believed to represent clinically elevated levels of general psychological distress, while a score of 10–12 on the depressive mood domain is believed to represent probable depression, and a score of 8 on the anxiety domain is believed to represent probable anxiety disorder [24]. The DASS-21 stress scale is believed to be sensitive to levels of chronic non-specific arousal, and was not explored in this study for its capacity to detect MDD.

The Impact of Events Scale-Revised (IES-R) is a 22-item self-report measure of trauma-related distress [31], validated in people with traffic injuries [30]. Respondents are asked to indicate their degree of distress during the past 7 days related to their recent road crash. It is a 5-point scale ranging from 0 (not at all) to 4 (extremely) for subscales avoidance (e.g. avoidance of feelings or situations), intrusion (e.g. intrusive distressing thoughts, nightmares), and hyperarousal (e.g. anger, irritability, hypervigilance). Domains are scored by determining the mean item score [31]. High scores indicate increased distress. Based on IES-R norms, a total score of 33 is believed to represent probable PTSD [33]. The IES-R has sound psychometric properties including acceptable reliability and validity [31, 33].

DSM-5 criteria for MDD and PTSD were used as a benchmark for determining the sensitivity and specificity of the DASS-21 and IES-R. For a positive MDD diagnosis, the participants needed to have reported at least five of the following DSM-5 criteria [37] with respect to their MVC experience (i) consistently depressed or down, most of the day, nearly every day for the past 2 weeks; (ii) much less interested in most things or much less able to enjoy the things they used to enjoy most of the time in the past 2 weeks; (iii) unintentional weight loss or gain; (iv) sleep difficulties (trouble falling asleep, frequent waking or waking very early); (v) agitation, restlessness, difficulty sitting still, talking more slowly; (vi) fatigued or loss of energy nearly every day; (vii) feeling worthless and guilty nearly every day; (viii) difficulty concentrating or making decisions almost every day, and (ix) frequent thoughts of death or suicidal ideation. MDD was then diagnosed if these symptoms have caused significant distress, have impaired their functionality, such as their ability to work or engage socially, and if the episode is not attributable to other conditions such as bereavement or substance abuse.

For a positive PTSD diagnosis, participants needed to report that they reacted with intense fear, helplessness or horror to the recent MVC in which they were physically injured, thus satisfying the first requirement for a PTSD diagnosis [37]. They also needed to report at least one of the following: (i) intrusion symptoms, that is, re-experiencing the MVC in a distressing way: memories, dreams, and/or flashbacks; (ii) persistent avoidance of stimuli associated with the MVC that arouse distress such as memories of the MVC, external reminders such as people, objects, and places; (iii) negative changes in cognitions and mood associated with the MVC: trouble recalling events, difficulty concentrating, feeling detached, reduced interests, sadness; and (iv) hyperarousal symptoms: irritability, anger, easily startled, constantly on guard. A PTSD was then diagnosed if these symptoms have been present since the MVC and have caused significant distress, and impaired their functionality, such as their ability to work or engage socially. Using a similar strategy, it was also determined whether participants met DSM-5 criteria for an adjustment disorder, which involves the development of significant distress in response to the MVC that is out of proportion to its severity [37].

### Statistical analysis

Descriptive statistics and frequency analyses were generated for the socio-demographic variables. Rates of MDD and PTSD in the sample based on DSM-5 criteria were determined using frequency breakdowns and contingency analyses. The required sample size to detect true differences with 80% statistical power (2 groups, α = .05, moderate effect size of 0.3) was estimated to be 90 [38]. To investigate the criterion validity of the DASS-21 and IES-R for use in a MVC population engaged in compensation, multivariate one-way analyses of variance (MANOVA) were conducted. For the first MANOVA, participants were divided into those meeting and not meeting DSM criteria for MDD, with the dependent variables being the three DASS-21 domains and DASS total score. For the second MANOVA, participants were divided into those meeting and not meeting DSM-5 criteria for PTSD, with the dependent variables being the three IES-R domains and total score. Univariate ANOVA was then conducted to determine significant differences. Partial eta-squared (*η*^*2*^) effect size values are provided as an estimate of the size of the difference between the groups. A partial *η*^*2*^of around .03 is considered small, .13 is considered a medium difference and over .2 is considered a large and substantial difference [39]. Post hoc or retrospective statistical power of the tests is also provided.

To determine the capacity of the two psychometric scales for estimating probable MDD and PTSD, various cut-off points based on norms [24, 33] for these two scales were explored, and *Χ*^2^, odds ratios, sensitivity and specificity values calculated. For each cut-off point test exploration, participants were divided into two sub-groups, that is, those scoring ≥ to the cut-off point (detected as having psychological disorder), versus those < the cut-off score. The decision rule on what constitutes a superior cut-off score for estimating probable psychological disorder was based on the following: (i) historical clinical norms, (ii) a significant *X*^2^ and odds ratio, (iii) the highest possible sensitivity and specificity, (iv) the lowest false negative (FN) and if possible (v) the lowest possible false positive (FP). A low FN is considered a priority, that is, a high sensitivity, as effective treatments are available for MDD and PTSD [10, 23]. Therefore the priority is on detecting those who actually have a psychological disorder, thus avoiding a misdiagnosis of a true positive, and consequently not being able to offer suitable treatment. FPs are also an important issue, especially so for regulatory bodies and insurers, given that offering treatment to those who do not have a disorder may not only misuse clinical/public health resources and funds, but also inflate compensation costs unnecessarily. The following are also provided: positive predictive value (PPV) which is the probability that a participant with a positive screen truly has the psychological disorder (displayed as a percentage), and negative predictive value (NPV), the probability that a participant with a negative screen truly does not have the disorder (also displayed as a percentage). A positive likelihood ratio (LR+) is provided, which is the extent to which a positive test increases the likelihood that a participant has the disorder, and a negative likelihood ratio (LR-), the extent to which a negative test decreases the likelihood that a participant has the disorder. LRs greater than 1 suggest the likelihood of the disorder is high, with larger the number, the more convincing that the detection of the disorder is correct. LRs between 0 and 1 suggest the likelihood of the disorder is low, with an LR close to 0 being unlikely. LRs of around 1 suggest the test lacks diagnostic value [40].

The capacity of the scales to estimate probable psychological disorder will also be compared to the ability of other factors that may be viable strategies for detecting psychological disorder, such as perceived danger in the MVC, and pre-MVC psychological morbidity. Participants’ scores for perceived danger were divided into 2 subgroups, the first sub-group consisted of those reporting no or small perceived danger, and the second sub-group consisted of those reporting moderate, great and overwhelming perceived danger. For pre-MVC psychological morbidity, participants were divided into those reporting versus not reporting receiving psychological treatment and taking psychiatric medication prior to the MVC. All analyses were performed using Statistica Software (Version 12, Statsoft).

## Results

Table 2 shows rates of MDD and PTSD detected in the 109 participants when using DSM-5 criteria. The rate of MDD was substantial at 53.2% of the sample, while the rate of PTSD was 19.3%. A contingency analysis showed that all PTSD cases except one were also diagnosed with MDD (*X*^2^ = 18.4, *df* = 1, *P* < .001; odds ratio: 26.3, 95% CI = 3.4 to 204.9, *P* < .001). In addition, all those diagnosed with an adjustment disorder (*n* = 14) except one met DSM-5 criteria for MDD (*X*^2^ = 9.9, *df* = 1, *P* < .01; odds ratio: 14.2, 95% CI = 1.8 to 112.6, *P* < .05). There was a less clear relationship between PTSD and adjustment disorder.

**Table 2:** Rates of major depressive disorder (MDD) and post-traumatic stress disorder (PTSD) in the 109 participants using the DSM-5 criteria

DSM-5 Diagnosis	MDD	PTSD
Yes	58 (53.2%)	21 (19.3%)
No	51 (46.8%)	88 (80.7%)

Results of the one-way MANOVA for DASS-21 indicated a significant difference as a function of the presence of MDD versus no MDD: Wilks lambda = .70, *F*_3,105_ = 14.8, *P* < .001, *η*^*2*^ = .30, power = 99.9%. In all cases, the DASS-21 scores were significantly higher (*P* < .001) for those with diagnosed MDD (see Table 3; large effect sizes of *η*^*2*^ > 0.2 were found for all four tests). Results of the one-way MANOVA for IES-R indicated a significant difference as a function of the presence of PTSD: Wilks lambda = .72, *F*_3,105_ = 13.2, *P* < .001, *η*^*2*^ = .27, power = 99.9%. In all cases, the IES-R scores were significantly higher (*P* < .001) for those with diagnosed PTSD (see Table 4; large effect sizes of *η*^*2*^ > 0.2 were found for the four tests).

**Table 3:** One-way MANOVA results for DASS-21 scores for those diagnosed or not diagnosed with MDD

DASS-21 domains and total score	MDD sub-group Mean (SD) 95% CI (*n* = 58)	No MDD sub-group Mean (SD) 95% CI (*n* = 51)	Total sample Mean (SD) 95% CI (N = 109)
Depressive mood	19.5 (13.1) 16.0–22.9	6.7 (8.2) 4.4–9.0*	13.5 (12.7) 11.1–15.9
Anxiety	16.9 (12.3) 13.7–20.2	6.1 (7.7) 3.9–8.2*	11.9 (11.7) 9.6–14.1
Stress	23.9 (12.1) 20.7–27.1	10.0 (9.6) 7.3–12.7*	17.4 (12.9) 14.9–19.6
Total score	60.3 (34.0) 51.4–69.3	22.8 (23.3) 16.3–29.4*	42.8 (34.8) 36.2–49.4

*P < .001

**Table 4:** One-way MANOVA results for IES-R scores for those diagnosed or not diagnosed with PTSD

IES-R domains and total score	PTSD sub-group Mean (SD) 95% CI (*n* = 21)	No PTSD sub-group Mean (SD) 95% CI (*n* = 88)	Total sample Mean (SD) 95% CI (*N* = 109)
Intrusion	25.1 (7.2) 21.8–28.4	12.9 (8.3) 11.1–14.7*	15.3 (9.4) 13.5–17.1
Avoidance	19.7 (7.1) 16.4–22.9	10.6 (7.7) 8.9–12.2*	12.4 (8.4) 10.8–13.9
Hyperarousal	18.5 (5.0) 16.2–20.7	9.7 (6.4) 8.3–11.0*	11.4 (7.0) 10.1–12.7
Total score	63.3 (17.7) 55.2–71.3	33.2 (20.3) 28.9–37.5*	39.0 (23.1) 34.6–43.4

*P < .001

Table 5 presents results of the sensitivity and specificity analyses for the cut-off scores for DASS-21. For the valid detection of probable MDD, and using the decision rule discussed in the Method, the following is recommended: (i) the DASS-21 total cut-off score of 30 can be applied to detect MDD, given it detected over 75% of actual MDD cases and around 70% of those not having MDD (PPV: 75.0%, NPV: 73.5%; LR+: 2.6; LR-: 0.3). This score is proposed as the optimal cut-off score to detect MDD. (ii) The DASS-21 depression domain could also be applied if a score of 10 is used, with over 75% of actual MDD cases detected and around 70% of those not having MDD detected (PPV: 72.4%, NPV: 74.5%; LR+: 2.6; LR-: 0.3). It is not recommended to apply the DASS-21 anxiety domain to detect MDD as its performance is inferior to the DASS-21 total and depression cut-off scores.

**Table 5:** True positive and negatives (TP, TN), false positive and negatives (FP, FN), chi-square (*X*^2^) results, odds ratios (OR), sensitivity (%) and specificity (%) results for DASS-21 total, depression and anxiety cut-off scores for probable MDD

Cut off score	TP	TN	FN	FP	*X* ^2^	OR	95% CI	Sensitivity	Specificity
Total score									
30	45	36	15	13	25.4**	8.3**	3.5–19.7	77.6	70.6
31	43	37	14	15	23.7**	7.6**	3.2–17.7	74.1	72.5
32	43	37	14	15	23.7**	7.6**	3.2–17.7	74.1	72.5
33	42	39	12	16	25.9**	8.5**	3.6–20.3	72.4	76.5
Depression									
10	42	38	13	16	23.9**	7.7**	3.3–18.0	76.4	70.4
11	40	39	12	18	22.5**	7.2**	3.1–16.9	76.9	68.4
12	40	39	12	18	22.5**	7.2**	3.1–16.9	69.0	76.5
13	34	43	8	24	21.1**	7.6**	3.0–19.1	58.6	84.3
Anxiety									
6	42	32	19	16	13.6*	4.4*	1.9–9.9	72.4	62.7
7	42	35	16	16	18.4**	5.7**	2.5–13.1	72.4	68.6
8	42	35	16	16	18.4**	5.7**	2.5–13.1	72.4	68.6
9	38	39	12	20	19.3**	6.2**	2.6–14.3	65.5	76.5

* *P* < .01 **P < .001; 95% CI: 95% confidence intervals for OR

Table 6 presents results of the sensitivity and specificity analyses for the cut-off scores for total IES-R and DASS-21 anxiety domain. Only the total IES-R was explored given the three domains all contribute to risk of PTSD. For the valid detection of probable PTSD, and using the decision rule discussed in the Method, the following is recommended: (i) the IES-R total cut-off score of 40 should be applied to detect PTSD, detecting over 90% of actual PTSD cases and from 61% of those not having PTSD (PPV range: 30.2–35.8%; NPV range: 95.6–96.4%; LR+ range: 1.8–2.3; LR- range: 0.19–0.16). Based on the decision rule, this score is therefore proposed as the optimal cut-off score to detect PTSD. (ii) The DASS-21 anxiety domain could also be applied if a cut-off score of 7 or 8 was used, with around 90% of actual MDD cases detected and around 50% of those not having MDD being detected (PPV: 32.7%; NPV: 96.1%; LR+: 2.0; LR-: 0.17).

**Table 6:** True positive and negatives (TP, TN), false positive and negatives (FP, FN), chi-square (*X*^2^) results, odds ratios (OR), sensitivity (%) and specificity (%) results for IES-R total cut-off scores for probable PTSD and DASS-21 anxiety domain

Cut off score	TP	TN	FP	FN	*X* ^2^	OR	95% CI	Sensitivity	Specificity
IES-R									
Total score									
32	19	43	45	2	10.8*	9.1*	2.0–41.3	90.5	48.9
33	19	44	44	2	11.4*	9.5*	2.1–43.2	90.5	50.0
34	19	46	42	2	12.6*	10.4*	2.3–47.4	90.5	52.3
35	19	47	41	2	13.2**	10.9*	2.4–49.6	90.5	53.4
36	19	50	38	2	15.2**	12.5*	2.7–57.0	90.5	56.8
40	19	54	34	2	18.2**	15.1**	3.3–68.9	90.5	61.4
DASS-21									
Anxiety									
6	19	46	42	2	12.6**	10.4*	2.3–47.4	90.5	52.3
7	19	49	39	2	14.5**	11.9*	2.6–54.4	90.5	55.7
8	19	49	39	2	14.5**	11.9*	2.6–54.4	90.5	55.7
9	17	55	33	4	12.9**	7.1*	2.2–22.8	80.1	62.5

* *P* < .01 **P < .001; 95% CI: 95% confidence intervals for OR

Note: IES-R cut-offs below 32 produce increased FN. IES-R cut-offs above 36 continue to produce reduced FP, but are becoming distant from the historical recommendation norm of 33

**Fig. 1:**
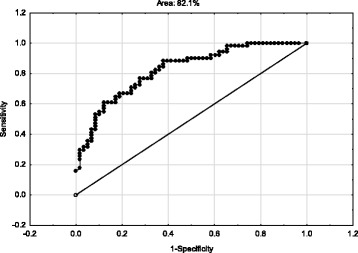
ROC curve showing the capacity of the DASS-21 (total, depression and anxiety scores) to detect MDD versus no MDD

**Fig. 2:**
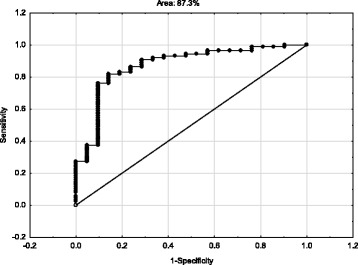
ROC curve showing the capacity of the IES-R (total scores) to detect PTSD versus no PTSD

Figures 1 and 2 show receiver operating characteristic (ROC) curves. The ROC plots the true positive rate (sensitivity) against the false positive rate (1-specificity) for detecting people who have probable MDD using the DASS-21 (only total, depression and anxiety scores) and probable PTSD using the IES-R (only total scores). Inspection of the Figures shows that in both cases the area under the curve was over 80% (82.1% and 87.3% for DASS-21 and IES-R respectively).

Overall, 29.4% (*n* = 32) had been treated by a psychologist or psychiatrist prior to the MVC, and 25.7% (*n* = 28) had taken psychiatric medications prior to the MVC. Neither strategy significantly detected MDD or PTSD, producing non-significant *X*^2^ and odds ratios (*P* > .05). For perceived danger, 56.9% (*n* = 62) perceived they were in at least moderate danger of death in the MVC. Perceived danger was not a significant strategy for detecting MDD with non-significant *X*^2^ and odds ratios (*P* > .05). However, perceived danger in the MVC did significantly detect PTSD (*X*^2^ = 6.1, *df* = 1, *P* < .05; odds ratio = 4.1, 95% CI = 1.2647 to 13.0412, *P* < .05, TP = 17, TN = 43, FP = 45, FN = 4, sensitivity = 80.9%, specificity = 48.9%; PPV: 27.4%; NPV: 91.5%; LR+: 1.6; LR-: 0.39).

## Discussion

Prior studies have shown that physical injury and psychological disorder associated with a MVC can have debilitating and long-lasting impacts on wellbeing [3, 13, 15]. The subsequent impairment and complications will substantially reduce personal capacity to be autonomous and restrict engagement in social and vocational activities. Accordingly, prior research has suggested that groups accounting for the highest percentage of injury costs should be targeted in health policy initiatives [41]. The cross-sectional findings from this study of baseline data from compensation claimants support the above assertion. In addition to the impact of physical injury, the sample showed high rates of psychological disorder when assessed at a mean of 11 weeks after the MVC. Over 50% of the sample received a diagnosis of MDD, while almost 20% were diagnosed with PTSD, and further, almost all those with a PTSD also had a co-morbid MDD (the odds of having PTSD if one had MDD was around 26:1). In addition, many with MDD also met DSM-5 criteria for adjustment disorder. These results are not dissimilar to the rates of MDD and PTSD found by prior research [15, 16]. However, there is evidence that the high rates of psychological disorder are not just a consequence of the MVC and physical injury, but also due to a dissatisfaction and distress associated with the compensation process [17, 42]. For example, based on prospective research, it was concluded that distress experienced when engaged in compensation following injury (mostly due to a MVC) was significantly related to disability in the long-term, and psychological disorder (e.g. trauma distress and depressive symptoms) increased distress experienced during the claims process, arguably leading to greater risk of more serious long-term disability [42]. It was further concluded that interventions delivered early after the injury that target those with elevated distress during compensation may improve physical and mental health and decrease compensation scheme timeframes and costs [42].

The results of the one-way MANOVA and the data shown in Table 3 indicate that the DASS-21 (total, depression, anxiety and stress) has excellent criterion validity for use in a MVC-related physically injured population engaged in compensation. Differences (e.g. effect sizes) between those with and without diagnosed MDD (using DSM-5 criteria) were significant and large. Similarly, the results of the one-way MANOVA (see Table 4) indicate that the IES-R (total score) also has excellent criterion validity for use in a MVC-related physically injured population engaged in compensation. Differences (e.g. effect sizes) between those with and without diagnosed PTSD (using DSM-5 criteria) were significant and large. These findings for DASS-21 and IES-R indicate both scales have excellent criterion validity when used with injured adults engaged in compensation. Furthermore, Figs. 1 and 2 support this conclusion. The area under the ROC curves was over 80% for each scale suggesting they can be validly and reliably used in public health and compensation contexts [25–28]. Used judiciously, the ROC curves suggest both scales have excellent potential for detecting injured people engaged in compensation who are at risk of psychological disorder [43].

However, a considerable problem still exists when using these two scales to achieve reliable detection of psychological disorder. Past research has provided clinical norms, but none have been provided for use with injured adults engaged in compensation [28, 32]. Therefore, the cut-off scores based on clinical norms and explored for their sensitivity and specificity when detecting MDD and PTSD, provide clarity about their capacity to detect disorder. It is recommended that a DASS-21 total cut-off score of 30 can be applied to detect MDD with acceptable sensitivity and specificity, while the DASS-21 depression domain cut-off score of 10 could also be applied, with acceptable sensitivity, specificity, high PPV and NPV, and LR+ and LR- values indicating appropriate likelihood of detection. Further, it is recommended that an IES-R total cut-off score of 40 can be applied to detect PTSD with excellent sensitivity and reasonable specificity. Cut-off scores up to 40 reduce FPs, though it is not recommended to apply cut-off scores over 40, as they are becoming distant from the historical norm of 33 [33]. The DASS-21 anxiety domain could also be applied if a cut-off score of 7 or 8 was used, with good sensitivity and specificity. Again, PPV and NPV percentages were acceptable for IES and DASS-21 anxiety domain, and LR+ and LR- indicated they have an appropriate likelihood of detection.

Nonetheless, a difficulty still remains. Regardless of whether a gold standard interview for MDD and PTSD or a self-report scale is used with recommended cut-off scores, errors of detection/diagnosis will always occur. Unquestionably, the goal is to reduce the frequency of diagnostic errors for both clinical and public health cost reasons. To achieve this, cut-off scores in the mild to moderate range were explored for DASS-21 and IES-R. Using the recommended cut-off scores for the DASS-21 and the IES-R will result in errors of detection (i.e. FNs and FPs). We believe the priority should be on optimizing the detection of those who actually have a psychological disorder, avoiding a misdiagnosis of a true positive. It is therefore proposed that for those scoring close to but below the cut-off score, there is some justification to conduct further assessment, such as referral to clinically trained professional for gold standard interviews. Further research will need to clarify how far below the recommended cut-off score remains a concern for further assessment, though we suggest assessing those falling within a 5–10% percentile below the cut-off score. For FPs, it is recommended that all those scoring above the accepted cut-off score should receive treatment. Such a strategy will ultimately reduce compensation and health costs [20].

The study has several limitations. A possible limitation concerns the inability to non-randomly select recruitment sites given the low number of potential sites in NSW and VIC (for instance in VIC there is only 1 site). The 109 participants are likely a biased sample given it is relatively small and that all participants were engaged in compensation. Also, the recruitment style used will result in bias, as well as the potential restrictions enforced by the exclusion/inclusion criteria. Any research that has an opt-in recruitment approach will have bias problems. Possible biases would include under-estimation of rates, for example distressed people may not want to participate due to low perceived benefits or over-estimation of rates, for example perhaps participants with higher distress could be more likely to participate in an intervention trial. The impact of these limitations on the occurrence rates of MDD and PTSD in the sample needs to be considered. Further, recruitment into the RCT has been slow, resulting in, at the present time, a sample of 109 participants at baseline. This is due in part, to the reluctance to participate in prospective research involving treatment soon after a MVC, especially in the context of the distress of a physical injury and engagement in a potentially stressful compensation process. However, the power analysis indicated the study had achieved 80% power with 90 participants. Achieved power in the study with 109 participants was estimated to be acceptable at 87% ensuring reduced Type II error rates [38]. The study will also be limited by up to 30% of the sample reporting they had psychological problems pre-MVC (i.e. seeing a psychiatrist/psychologist and taking psychotropic medications). This could potentially increase numbers having MDD and to a lesser degree PTSD, however, these variables were not significant detectors of MDD or PTSD post-MVC. A limitation exists with regard to the benchmark rating employs DSM-5 criteria via online self-report. Whilst acknowledging that the gold standard rating for DSM-5 clinical interview is based on face to face clinical assessment by a trained professional, evidence suggests computer assisted self-report strategies are effective for diagnosis [44] and further, substantial information in our diverse suite of assessments were available if a diagnosis of MDD or PTSD required clarification. In regard to the screening tools, there are several other avenues for determining validity and reliability such as test-retest, split-half and alternate forms procedures. This study only investigated criterion validity.

## Conclusions

The costs associated with managing disability following MVC-related injury in people with psychological disorder will be substantial if no action is taken to address this problem [20]. This is a concern from a public health perspective. The data demonstrate that by 11 weeks post-MVC, potentially over 50% of injured adults will meet DSM-5 criteria for MDD, and almost 20% will meet criteria for PTSD. This is a high rate of psychological disorder whose impacts could well continue into the longer-term, increasing chances of significant disability. At risk is increased poor social re-integration, delayed return to work and consequent reduced quality of life [10]. Through the establishment of criterion validity for screening psychological disorder in a compensation MVC-injured population, the findings of this study provide potentially reliable benchmarks for determining the need for psychological intervention in people sustaining injury in a MVC and engaged in compensation. People who screen positively can be referred to appropriately and clinically trained professionals for further assessment, and if this also proves positive, the person can then be provided with information about appropriate and available evidenced-based treatment. Judicious use of scales like the DASS-21 and IES-R to detect rates of psychological disorder will hopefully contribute to improved outcomes. For instance, use of these two sensitive instruments will be economical compared to using expensive DSM based psychiatric interviews, and lead to prudent recommendations by insurers for appropriate and if possible, early intervention for those at risk. It is hoped the data will have the potential to influence public health decisions in injury management. It is also anticipated this study will help clarify for insurance companies and clinicians what constitutes severe psychological disorder from mild to moderate psychological distress. The findings will also enhance rehabilitation of people injured in MVCs as it will not only assist in the diagnosis of people with psychological disorder but also these findings will hopefully lead to effective brief psychological interventions designed to prevent psychological disorder from occurring when delivered as soon after the MVC as possible.

## Abbreviations

DASS: Depression Anxiety Stress Scales
IES-R: Impact of Events Scale Revised
MDD: Major depressive disorder
MVC: Motor vehicle crash.
PTSD: Post traumatic stress disorder
